# Comparative genomic surveillance of carbapenem-resistant *Acinetobacter baumannii* in the Netherlands in 2015–2017 and 2022–2024

**DOI:** 10.1128/spectrum.02602-25

**Published:** 2026-02-26

**Authors:** Max P. Allewijn, Sandra Witteveen, Karuna E. W. Vendrik, Angela de Haan, Fabian Landman, Jeroen Bos, Simon Lansu, Cornelia C. H. Wielders, Annelot F. Schoffelen, Sabine C. de Greeff, Daan W. Notermans, Antoni P. A. Hendrickx, On behalf of the Dutch CPE Surveillance Study Group

**Affiliations:** 1 Centre for Infectious Disease Control (CIb), National Institute for Public Health and the Environment (RIVM) 10206, Bilthoven, the Netherlands; Rowan University Cooper, Camden, New Jersey, USA

**Keywords:** antimicrobial resistance, Ukraine, CRAb, *Acinetobacter baumannii*, carbapenem resistance, the Netherlands, genomics, surveillance, NDM-1, NDM-5

## Abstract

We investigated the genomic epidemiology of carbapenem-resistant *Acinetobacter baumannii* (CRAb) during two surveillance periods: 2015–2017 (pilot 1) and 2022–2024 (pilot 2) in an international context to identify potential circulating high-risk lineages in the Netherlands. A total of 204 CRAb isolates were all analyzed by the carbapenem inactivation method, meropenem Etest, and whole-genome sequencing (combining Illumina and Nanopore). Comparative resistome, multilocus sequence typing (MLST), and whole-genome MLST (wgMLST) analyses were performed between pilots and compared to 577 international CRAb genomes. The Dutch CRAb population remained genetically diverse and stable between pilots and was dominated by international clone (IC)2 (61.8%), followed by IC9 (11.3%), IC1 (6.4%), and IC6 (5.9%). Also, epidemiological and clinical characteristics remained stable across both pilots. In contrast, CRAb in patients from Ukraine was genetically diverse and an epidemiologically distinct group. wgMLST indicated an increase in genetic clustering in 2022–2024, with unique CRAb clusters associated with Ukrainian patients. Of 26 identified clusters, 69% were linked to international sources. Differences in resistomes were observed between CRAb in patients from the Netherlands and Ukraine, and between pilots. Overall, 75% of isolates harbored *bla*_OXA-23_-like genes, and *bla*_NDM_ genes increased from 16% to 21%. None of the CRAb isolates from Ukrainian patients carried *bla*_NDM_ genes; instead, they harbored *bla*_OXA-23_-like and *bla*_OXA-24_-like genes. Hybrid assemblies showed that carbapenemase genes were predominantly chromosomal. In conclusion, CRAb in the Netherlands showed genetic stability over time, yet the presence of New Delhi metallo-β-lactamase-carbapenemases and import of strains from high-risk regions underline the necessity for ongoing national surveillance.

**IMPORTANCE:**

In 2015–2017 and 2022–2024, pilot surveillance of carbapenem-resistant *Acinetobacter baumannii* (CRAb) was performed in the Netherlands. Three distinct groups were compared based on epidemiological and genomic data: CRAb from 2015 to 2017 and for the 2022–2024 period, CRAb in patients from the Netherlands, and CRAb in patients from Ukraine in the Netherlands. The genomic epidemiology of CRAb has been mostly stable between 2015–2017 and 2022–2024 but seems to be strongly affected by the introduction of CRAb from foreign countries. A proportion of the 2022–2024 isolates were from Ukrainian patients. The resistance genes of CRAb from Ukrainian patients were different from those of the Netherlands. CRAb in the Netherlands are acquiring New Delhi metallo-β-lactamase-carbapenemase genes, aided by the introduction of CRAb from foreign countries. Healthcare professionals should remain aware of the risk of hospitalization in a high-risk country and the potential secondary transmission of CRAb to patients in countries with a low prevalence of this organism.

## INTRODUCTION

Bacteria belonging to the *Acinetobacter baumannii-calcoaceticus* complex (ABC-complex) are gram-negative coccobacilli that have emerged over the last decades as a cause of healthcare-associated infections and, to a lesser extent, of community-acquired infections (1). The ABC-complex includes the closely related species: *A. baumannii*, *A. calcoaceticus*, *A. nosocomialis*, *A. pittii*, *A. seifertii*, and *A. lactucae. A. baumannii* is by far the most common species and can cause nosocomial urinary tract, bloodstream, respiratory tract, and wound infections (2–4).

Reports of carbapenem resistance in *A. baumannii* date back to the early 1990s (5, 6). In recent years, however, *A. baumannii* has gained importance, as carbapenem-resistant *A. baumannii* (CRAb) was listed first by the WHO in 2017 as a critical antibiotic-resistant pathogen posing the greatest threat to human health (7), and in the updated list of 2024, CRAb still ranked in the top three (8). In multidrug-resistant CRAb strains, colistin is often used as a last-resort treatment; however, colistin-resistant clinical CRAb isolates have already been reported, thereby complicating antimicrobial therapy (9–12)*.*

*A. baumannii* carries oxacillinases (OXA) and New Delhi metallo-β-lactamases (NDM) associated with specific multilocus sequence typing (MLST) sequence types (ST). *A. baumannii* lineages have been characterized, termed international clones (ICs), that originated and disseminated in different parts of the world (13–15). While the Netherlands has a relatively low rate of antimicrobial resistance among *A. baumannii* (16), surveillance of CRAb is important to monitor emerging STs, carbapenemases, and other antimicrobial resistance genes and to detect possible transmission events within and between hospitals in order to prevent further nosocomial spread. Whole-genome sequencing (WGS) enables genomic surveillance of CRAb (17). By identifying nosocomial transmission events, hospitals can be informed and take further preventative infection prevention action. Additionally, by identifying the prevalent CRAb STs and emerging antibiotic resistance patterns, national treatment guidelines can be evaluated more effectively. However, relatively little is known about the molecular characteristics and spread of CRAb in the Netherlands. In 2015–2017, a nationwide pilot surveillance of carbapenemase-producing *A. baumannii* (CPAb) was performed, but the surveillance was discontinued. Due to renewed attention for CRAb by the WHO, another CRAb pilot surveillance was performed in 2022–2024. In both pilot surveillances, CRAb isolates were collected using the existing national surveillance network infrastructure covering all Dutch hospitals (17). CRAb isolates were from cultures for diagnostic reasons as well as from screening for carriership with carbapenemase-producing microorganisms, which is standard practice upon admitting patients with a recent hospitalization abroad. The major aim of this study was to compare the genomic epidemiology of CRAb in 2015–2017 and 2022–2024 in the Netherlands.

## RESULTS

### Characteristics of patients carrying *A. baumannii*, the Netherlands, 2015–2017 and 2022–2024, *n* = 204

In the two pilots, *n* = 207 isolates from *n* = 207 patients were selected belonging to the ABC complex with an MIC > 8 mg/L for meropenem. Out of these isolates, 204 were determined to be *A. baumannii* after analysis with KRAKEN2*.* Three isolates (*A. nosocomialis* and *A. pittii*) were excluded from the data set, resulting in a total of *n* = 204 isolates from 204 patients used for analysis (see below). For pilot 1, the National Institute for Public Health and the Environment (RIVM) received *n* = 83 *A. baumannii* isolates, and for pilot 2, the RIVM received *n* = 121 *A. baumannii* isolates*.* In pilot 2, the majority of 97/121 *A. baumannii* isolates were obtained from patients residing in the Netherlands, and 24/121 isolates were obtained from patients who originated from Ukraine and were transferred to the Netherlands for treatment.

Table 1 provides a summary of the clinical and epidemiological data collected from the patients carrying CRAb in these three groups. For patients from the Netherlands in both pilots 1 and 2, there was a wide age range (2–87 and 17–92 years), with the median age being 69 and 71 years, respectively. The median age for patients from Ukraine was 36 years old (range 19–66 years). Most patients were male in all groups (69% for pilot 1, 64% for pilot 2 patients from the Netherlands, and 96% for pilot 2 patients from Ukraine). Roughly half of the isolates were obtained from screening swabs (45% for pilot 1, 54% for pilot 2 patients from the Netherlands, and 46% for pilot 2 patients from Ukraine), with wounds and urine being the most common material of origin among diagnostic samples. Wounds represented 29% of the sample materials of CRAb from Ukrainian patients. Of the total number of patients from the Netherlands from pilots 1 and 2, 27% and 39% were hospitalized abroad in the 2 months prior to sampling, respectively. For patients from Ukraine, 67% were hospitalized in a foreign hospital in the 2 months prior to sampling, often related to the war in Ukraine. For the remainder of patients, no data on previous foreign hospitalization were provided. The most common countries of recent foreign visits or hospitalization among patients from the Netherlands were Morocco (*n* = 19), Greece (*n* = 14), and Türkiye (*n* = 9).

**TABLE 1:** Characteristics of patients with carbapenem-resistant *A. baumannii* isolates collected in the Netherlands, pilot 1 (2015–2017), and pilot 2 (2022–2024 divided in patients residing in the Netherlands [Dutch patients] and from Ukraine [UA patients]), *n* = 204^*a*^

Group	Pilot 1 2015–2017, *n* = 83		Pilot 2 2022–2024 Dutch patients, *n* = 97		Pilot 2 2022–2024 UA patients, *n* = 24		Total, *n* = 204	
				
Median age (first to third quartile)	69	57–76	71	60–77	36	28–42	69	49–76
Gender	*n*	%	*n*	%	*n*	%	*n*	%
Male	56	69%	62	64%	23	96%	141	70%
Female	25	31%	35	36%	1	4%	61	30%
No data available	2		0		0		2	
Provider of material	*n*	%	*n*	%	*n*	%	*n*	%
Hospital	47	57%	83	86%	24	100%	154	75%
Intensive care	9	11%	15	15%	0	0%	24	12%
Other inpatients	23	28%	28	29%	17	71%	68	33%
Outpatient	4	5%	2	2%	0	4%	6	3%
Unknown	11	13%	38	39%	7	25%	62	30%
General practitioner	2	2%	10	10%	0	0%	12	6%
Nursing home/elderly home/care center	1	1%	3	3%	0	0%	4	2%
Health service	0	0%	1	1%	0	0%	1	0%
Unknown	33	40%	0	0%	0	0%	33	16%
Material of origin	*n*	%	*n*	%	*n*	%	*n*	%
Swab of throat/nose/perineum/rectum	37	45%	52	54%	11	46%	100	49%
Wound/pus	14	17%	15	15%	7	29%	36	18%
Urine	9	11%	14	14%	0	0%	23	11%
Sputum	8	10%	7	7%	0	0%	15	7%
Bronchoalveolar lavage/aspirate	3	4%	0	0%	0	0%	3	1%
Tissue	1	1%	2	2%	2	8%	5	2%
Blood	1	1%	2	2%	1	4%	4	2%
Artificial material	1	1%	2	2%	2	8%	5	2%
Other	2	2%	2	2%	0	0%	4	2%
Unknown	7	8%	1	1%	1	4%	9	4%
Foreign visits/hospitalization	*n*	%	*n*	%	*n*	%	*n*	%
Hospitalized <2 months prior to sampling in a foreign hospital for >24 h	22	92%	43	100%	16	100%	81	98%
Hospitalized in a foreign hospital >2 months and <1 year prior to sampling	1	4%	0	0%	0	0%	1	1%
Visited a foreign country <6 months prior to sampling without visiting a hospital	1	4%	0	0%	0	0%	1	1%
No data available	59		54		8		121	
Country of foreign visit/hospitalization	*n*	%	*n*	%	*n*	%	*n*	%
Morocco	6	25%	13	30%	0	0%	19	22%
Greece	4	17%	10	23%	0	0%	14	16%
Türkiye	4	17%	5	12%	0	0%	9	11%
Thailand	3	13%	2	5%	0	0%	5	6%
Indonesia	2	8%	2	5%	0	0%	4	5%
Ukraine	0	0%	0	0%	16	100%	18	21%
Other^*b*^	5	21%	11	26%	0	0%	16	19%

^*a*^ UA, Ukrainian.

^*b*^ Countries with *n* ≤ 2 in total were grouped in the category “Other” (*n *= 14). Percentages are calculated based on available data.

### Genetic relation of CRAb isolates and putative transmission clusters, the Netherlands, 2015–2017 and 2022–2024, *n* = 204

**Fig 1:**
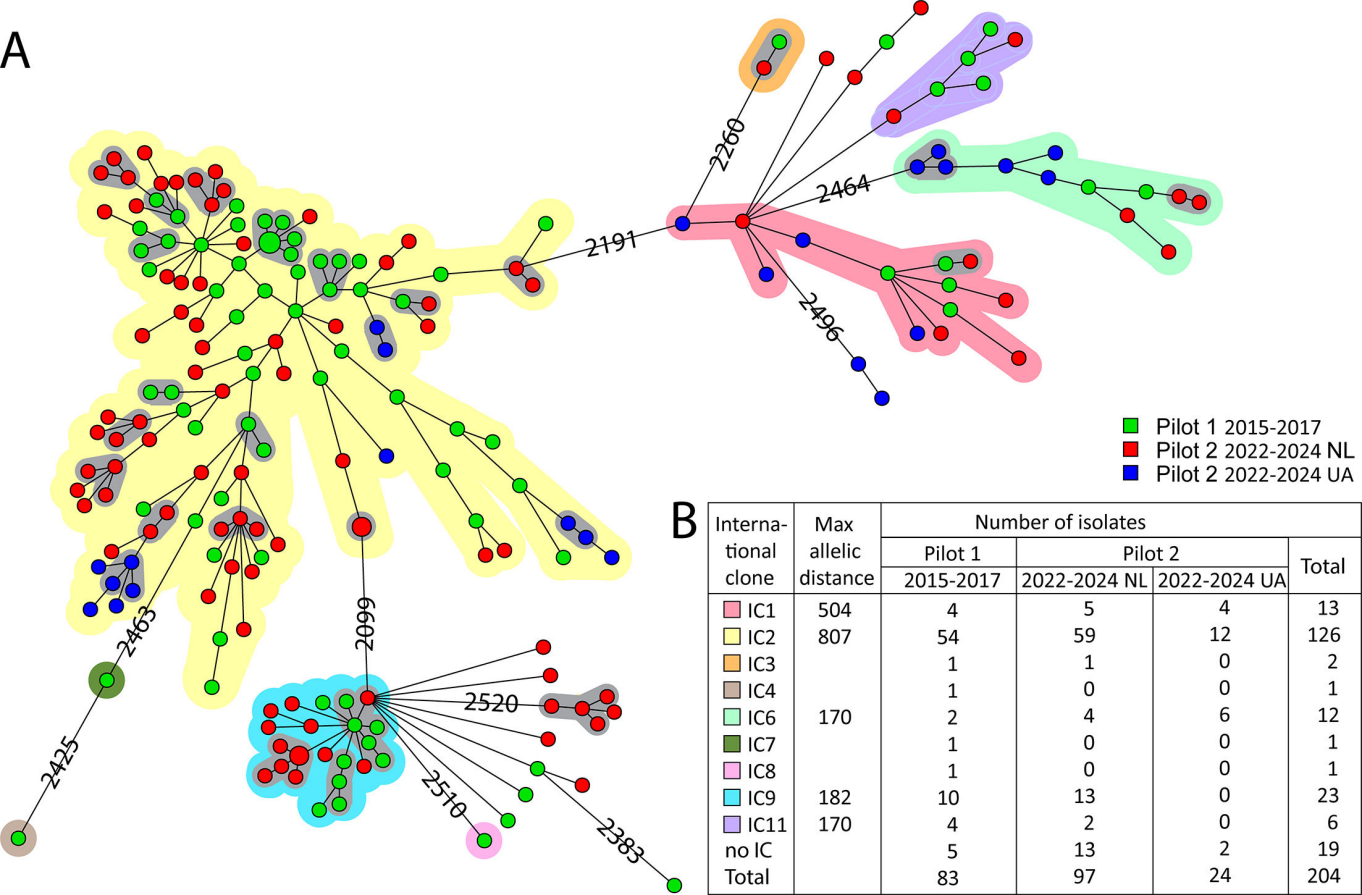
Genetic relation and putative transmission clusters visualized in a wgMLST-based MST of carbapenem-resistant *A. baumannii* from the Netherlands, 2015–2017 and 2022–2024, *n* = 204. (**A**) The CRAb isolates from patients from the Netherlands from pilot 1 are shown as green circles and from pilot 2 as red. The CRAb isolates found in patients associated with Ukraine from pilot 2 are blue. Clusters of ≥2 isolates that differ by ≤20 wgMLST ADs are highlighted with gray halos. Each smaller circle represents an isolate, and larger circles represent more than one isolate with identical wgMLST profiles. (**B**) Overview of the number of isolates belonging to known ICs. The ICs are highlighted in the MST tree by different colors: red, yellow, orange, brown, dark green, purple, light green, blue, and pink. The first column gives the maximum AD between any two isolates within the corresponding IC if the number of isolates is more than three.

The whole-genome MLST (wgMLST) profiles of the 83 CRAb isolates from pilot 1 and the 121 CRAb isolates from pilot 2 were compared (Fig. 1A). The CRAb isolates of both pilots 1 and 2 were randomly distributed in the minimum spanning tree (MST). Known MLST STs and ICs were assigned and matched in *bla*_OXA-51_-like variant content (15). Based on wgMLST, the designated ICs form groups of isolates that have an allelic difference (AD) between the IC groups of >1,000 AD. The majority of the CRAb isolates in this study belonged to IC2 (126/204; 61.8%) and to IC9 (23/204; 11.3%), followed by IC1 (13/204; 6.4%) and IC6 (12/204; 5.9%; Fig. 1B). Nineteen of 204 (9.3%) isolates did not group in a known IC. CRAb associated with Ukrainian patients belonged to IC1 (4/24; 17%), IC2 (12/24; 50%), and IC6 (6/24; 25%). The most common STs were ST195 (30 isolates, IC2), ST208 (19 isolates, IC2), and ST1089 (14 isolates, IC9). IC2 contained 32 different STs in total.

When using a cut-off value of ≤20 wgMLST AD to assign two isolates to a genetic cluster, *n* = 26 genetic clusters were observed (Table 2). Seven genetic clusters were observed among pilot 1 isolates, 15 clusters comprising pilot 2 isolates, and 4 genetic clusters containing isolates from patients from the Netherlands in both pilot 1 and pilot 2. Their respective STs can be found in Table 2. From pilot 2, 11 genetic clusters contained only isolates from patients from the Netherlands, and 4 clusters contained only isolates from Ukrainian patients. To date, there were no clusters containing isolates from both patients from the Netherlands and Ukraine. In 18/26 (69%) clusters, a part of the CRAb isolates originated from patients with a history of hospitalization abroad less than 1 year prior to sampling (Table 2); for the remaining clusters, no patient data on previous hospitalization abroad were available. Morocco (*n* = 5), Greece (*n* = 5), and Ukraine (*n* = 4) were the most common associated countries. The Ukraine-associated CRAb clusters contained only CRAb from Ukrainian patients. Genetic cluster-13, consisting of CRAb from four patients out of five who were previously hospitalized in Morocco, and the isolates did not belong to an existing IC (Table 2).

**TABLE 2:** The carbapenem-resistant *A. baumannii* genetic clusters found in the Netherlands, carbapenemase genes, MLST types, and country of previous hospitalization, 2015–2017 and 2022–2024, *n* = 204^*c*^

Genetic cluster	Reported IC	Number of isolates from pilot 1/pilot 2 NL/pilot 2 UA	Carbapenemase genes	MLST ST (Oxford)	Number of patients previously hospitalized in a foreign country^*a*^/out of total patients within the genetic cluster (country)
1	IC2	2/0/0	*bla*_OXA-23_	ST105	0/2 (No data)
2	IC9	5/1/0	*bla*_NDM-1_	ST1089	2/6 (Morocco)
3	IC2	3/0/0	*bla*_OXA-23_	ST218	1/3 (Greece)
4	IC9	3/0/0	*bla*_OXA-23_	ST1089	0/3 (No data)
5	IC2	2/0/0	*bla*_OXA-23_	ST208	0/2 (No data)
6	IC2	7/0/0	*bla*_OXA-23_	ST195	7/7 (Local Outbreak)^*b*^
7	IC2	2/0/0	*bla*_OXA-23_	ST208	0/2 (No data)
8	IC2	2/0/0	*bla*_OXA-23_	ST195	2/2 (Greece)
9	IC9	0/6/0	*bla*_NDM-1_	ST2782	4/6 (Morocco)
10	IC2	0/3/0	*bla*_OXA-23_	ST195	1/3 (Greece)
11	IC2	0/0/3	*bla*_OXA-23_	ST2063	3/3 (Ukraine)
12	IC2	0/2/0	*bla*_OXA-23_	ST2063	0/2 (No data)
13	No IC	0/5/0	*bla*_OXA-23_	ST732	4/5 (Morocco)
14	IC2	1/1/0	*bla*_OXA-23_	ST451	1/2 (Türkiye)
15	IC2	0/3/0	*bla*_OXA-23_	ST195	1/3 (Türkiye)
16	IC3	1/1/0	*bla*_OXA-23_	ST106	2/2 (Morocco)
17	IC2	0/2/0	*bla*_OXA-23_	ST425/ST3341	1/2 (Greece)
18	IC2	0/2/0	*bla*_OXA-23_	ST1841	1/2 (Morocco)
19	IC2	0/2/0	*bla*_NDM-1_ *, bla*_OXA-23_	ST1578	0/2 (No data)
20	IC6	0/2/0	*bla*_OXA-23_	ST1961	1/2 (Suriname)
21	IC1	1/1/0	*bla*_OXA-23_	ST405	0/2 (No data)
22	IC2	0/0/2	*bla*_OXA-23_	ST3345	2/2 (Ukraine)
23	IC6	0/0/3	*bla*_OXA-72_	ST3279	3/3 (Ukraine)
24	IC2	0/3/0	*bla*_OXA-23_	ST425	3/3 (Greece)
25	IC2	0/0/2	*bla*_OXA-23_	ST546/ST3310	2/2 (Ukraine)
26	IC2	0/3/0	*bla*_OXA-201_	ST208	1/3 (Canary Islands)

^*a*^ Patients hospitalized <2 months prior to sampling, based on 76 known answers as shown in Table 1.

^*b*^ The first isolate from this genetic cluster was a patient who had been hospitalized more than 24 h in Indonesia less than 2 months prior to sampling.

^*c*^ NL, the Netherlands; UA, Ukraine.

### Genomic comparison of the CRAb isolates from the Netherlands with international CRAb isolates

**Fig 2:**
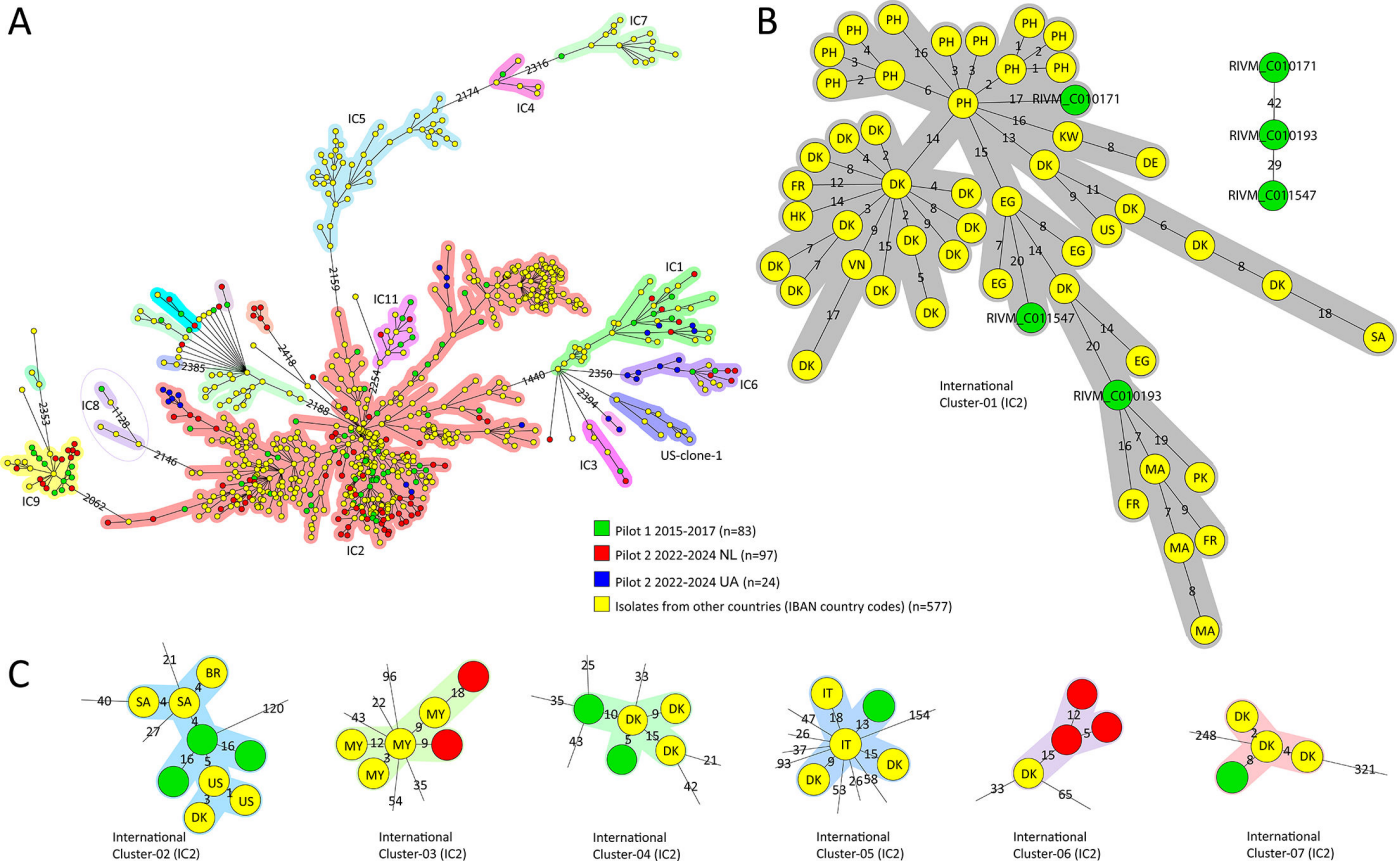
Genetic relation and clonal spread of carbapenem-resistant *A. baumannii* in the Netherlands *n* = 204 in context of *n* = 577 international CRAb, *n* = 781. (**A**) wgMLST-based MST of CRAb isolates from patients from the Netherlands of pilot 1 is in green and from pilot 2 is red. The CRAb isolates found in patients associated with Ukraine in pilot 2 are blue. In yellow are the international CRAb WGS sequences downloaded from NCBI. Groups of isolates that differ by >1,000 wgMLST ADs are highlighted with different colored halos representing the ICs. Each circle represents an isolate, and a larger circle represents >1 isolate without wgMLST AD. (**B**) The largest international genetic CRAb cluster containing three isolates from patients in the Netherlands (green) and 48 international (yellow) isolates from different countries indicated by ISO-3166 alpha-2 country codes. Next to the cluster are the three isolates depicted in green, showing that the AD is ≥20, not forming a genetic cluster. (**C**) Close-up of the genetic clusters with at least four isolates, containing both Dutch and international CRAb isolates, indicated by country codes. BR, Brazil; DE, Germany; DK, Denmark; EG, Egypt; FR, France; HK, Hong Kong; IT, Italy; KW, Kuwait; MA, Morocco; MY, Malaysia; PH, the Philippines; PK, Pakistan; SA, Saudi Arabia; US, the United States of America; VN, Vietnam.

To place the CRAb from patients in the Netherlands in an international context, the 204 CRAb isolates from the Netherlands from the two pilot surveillances were compared to 577 international CRAb sequences obtained from 12 different countries (Fig. 2A). By combining these data sets, the CRAb in patients from the Netherlands from pilot 1 and 2 grouped in internationally described ICs, except in IC5 and US-clone-1, which were not found in the Netherlands. The highest AD of 1128 was between two isolates within IC8, and the lowest AD of 1440 was between IC2 and IC1 (Fig. 2A). Fifteen genetic clusters of an isolate from a patient from the Netherlands and an international isolate were identified. Cluster-01 represented the largest genetic cluster containing 51/781 isolates (Fig. 2B), with three isolates from patients from the Netherlands from pilot 1 (2015–2017) and isolates from Denmark, the United States, the Philippines, Morocco, Egypt, France, Kuwait, Vietnam, Germany, Hong Kong, Saudi Arabia, and Pakistan. Without these international isolates, the CRAb in patients from the Netherlands did not cluster since the AD was 29–42; therefore, transmission within the Netherlands seems unlikely in this case. Six other smaller clusters are highlighted, each containing four isolates or more (Fig. 2C), while clusters with less than four isolates were not shown. In four of the smaller clusters, CRAb in patients from the Netherlands clustered together with isolates from Denmark. Three Danish isolates in Cluster-04 had a travel-related link with Greece (Cluster-11 in reference 13). The two isolates from the Netherlands in this cluster belonged to Dutch Cluster-08, and both patients had been hospitalized before in Greece. Three Danish isolates in Cluster-07 had a travel-related link with Türkiye (cluster 7 in reference 13), but no epidemiological data about the patients from the Netherlands were available. The two CRAb isolates from patients in the Netherlands in Cluster-03 did not form a cluster with each other without the international isolates.

### The resistomes of CRAb isolates in the Netherlands, 2015–2017 and 2022–2024, *n* = 204

All CRAb isolates had one or more resistance genes encoding for beta-lactam resistance. All isolates were positive in the carbapenem inactivation method (CIM) and had an MIC for meropenem of >8 mg/L as determined by E-test. A *bla*_OXA-23_-like gene was present in 75% (152/204) of isolates (Table 2). In pilot 1, 16% (13/83) of CRAb contained *bla*_NDM-1_, and in pilot 2, 21% (20/97) contained either a *bla*_NDM-1_ or *bla*_NDM-5_ carbapenemase gene. The *bla*_NDM-5_ carbapenemase gene was first detected in pilot 2. Notably, none of the 24 Ukrainian CRAb isolates contained *bla*_NDM_ genes, while these isolates were enriched with *bla*_OXA-23_-like/*bla*_OXA-24_-like genes. Of the 33 *bla*_NDM_-carrying isolates, 14 were from patients who had been hospitalized in a foreign country less than 2 months prior to sampling. In nine of these cases, there was a link to Morocco, twice to Thailand, and once to Egypt, Ghana, and Türkiye. The *bla*_NDM_*-*carrying isolates belonged to IC9 (*n* = 17), IC2 (*n* = 12), and IC8 (*n* = 1) and unknown IC (*n* = 3; File S1).

**Fig 3:**
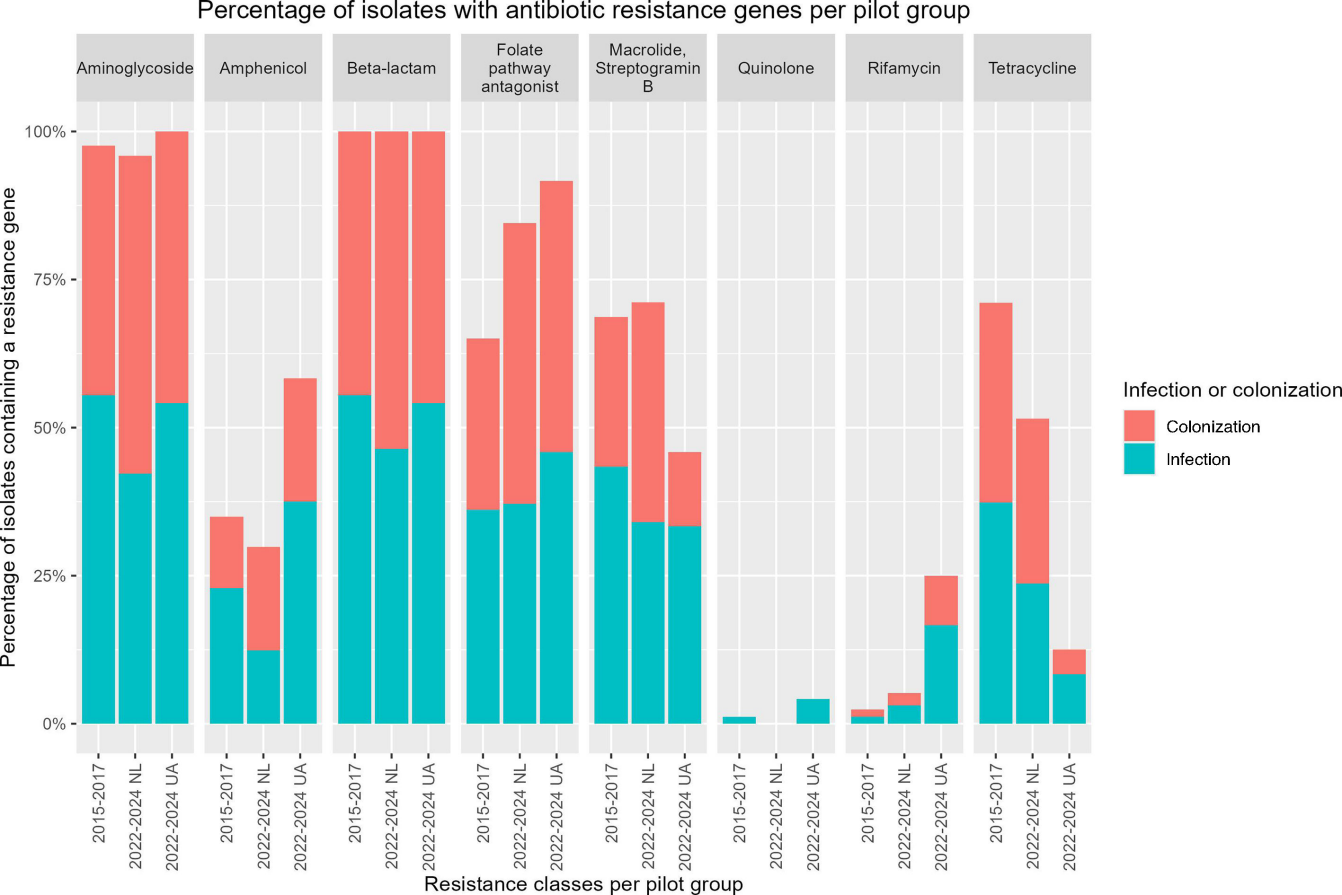
Bar plot of the prevalence of resistance genes in carbapenem-resistant *A. baumannii* in the Netherlands (NL), 2015–2017 and 2022–2024, *n* = 204. Data were generated with WGS, and resistance genes were identified using ResFinder. Antibiotic resistance genes were grouped by antibiotic class. Infection or colonization was assigned based on the sampling material (colonization for swabs of throat/nose/perineum/rectum, the remaining for infection). 2015–2017 pilot 1 (*n* = 83), 2022–2024 pilot 2 CRAb from patients from the NL (*n* = 97), and 2022–2024 pilot 2 CRAb from patients from Ukraine (UA, *n* = 24) for different antibiotic groups.

Based on eight different antibiotic classes, most isolates had predicted resistances to 4–5 antibiotic classes. This did not significantly differ between the three groups (one-way analysis of variance test; Fig. S1). More than 95% of the CRAb isolates had a gene encoding for aminoglycoside resistance (mainly an *aph(3'')* variant or *armA*; Fig. 3; File S1). The presence of genes encoding predicted resistance toward aminoglycosides, beta-lactams, macrolides, and streptogramin B was similar among CRAb isolates from patients in the Netherlands in both pilots (Fig. 3). The presence of resistance genes to folate pathway antagonists, such as sulfonamide, increased between pilots 1 and 2 and was highest among CRAb isolates from Ukrainian patients. This was entirely due to an increase in s*ul1* and s*ul2* genes, whereas resistance genes for trimethoprim remained rare (*n* = 17, File S1). In contrast, tetracycline resistance (mostly due to *tetB*) decreased between pilots 1 and 2 and was lowest among CRAb isolates from Ukrainian patients. Rifamycin resistance was rare in CRAb isolates from the Netherlands (2/83; 2% in pilot 1, and 5/97; 5% in pilot 2) but was more commonly found in Ukrainian isolates (6/24; 25%). (Fluoro-)quinolone resistance genes were found in one isolate in pilot 1 and one isolate from a Ukrainian patient. No *mcr* mobile colistin resistance genes were detected among any of the 204 isolates.

### Distribution of antibiotic resistance genes on carbapenem-resistant *A. baumannii* genomic elements

**Fig 4:**
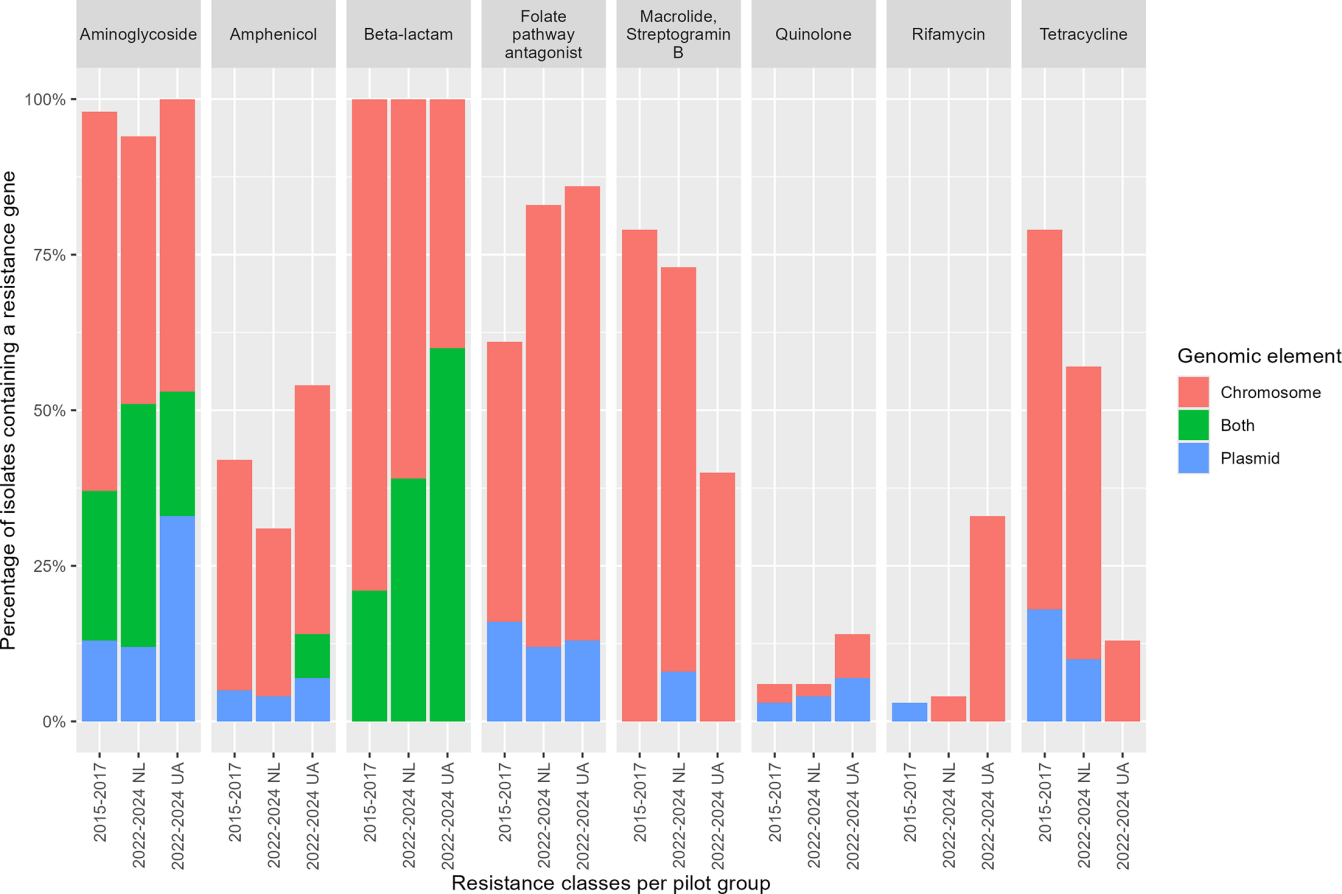
Bar plot of the prevalence of resistance genes on genomic elements in carbapenem-resistant *A. baumannii* in the Netherlands (NL), 2015–2017 and 2022–2024, *n* = 102. Data were generated with a hybrid assembly of short- and long-read WGS data and ResFinder. Antibiotic resistance genes were grouped by antibiotic class. Only isolates with a complete circular chromosome and plasmid were included. A genomic element was assigned based on if a resistance gene is present on only the chromosome, only the plasmid, or on both. The 102 hybrid assemblies were distributed over the pilots: 2015–2017 pilot 1 (*n* = 38), 2022–2024 pilot 2 CRAb from patients from the NL (*n* = 49), and 2022–2024 pilot 2 CRAb from patients from Ukraine (UA, *n* = 15).

Based on a hybrid assembly of short- and long-read WGS data, it was possible to identify whether resistance genes were present on the chromosome or on plasmids or both. For 102 out of 204 isolates, this resulted in the successful assembly of circular chromosomes and plasmids; linear fragments were not analyzed. For 6/8 of the antibiotic classes, the majority (417/547; 89%) of resistance genes were localized on the chromosome (Fig. 4). All isolates carried at least one *bla*_OXA_ gene (excluding *bla*_OXA-51_) on the chromosome, but when an isolate carried two *bla*_OXA_ genes, they were also found on plasmids (39/102; 38%). Between pilot 1 and 2, there was an increase in isolates carrying an aminoglycoside (9/38; 24% to 19/49; 39%) or beta-lactam resistance (8/38; 21% to 19/49; 39%) gene on both the chromosome and a plasmid. Of the 102 complete hybrid assemblies, 19 were *bla*_NDM_-positive, and 18 of the 19 *bla*_NDM_ genes were found on the chromosome. The increase of resistance genes for folate pathway antagonists was only found on the chromosome, not on plasmids.

## DISCUSSION

Two pilot surveillances were performed in the periods 2015–2017 (pilot 1) and 2022–2024 (pilot 2) to investigate the genomic epidemiology of carbapenem-resistant *A. baumannii* in the Netherlands. The genetic CRAb population was diverse and carried a variety of combinations of carbapenemases but remained relatively stable over the years 2015–2017 and 2022–2024 for patients from the Netherlands. The most dominant IC found in the Netherlands was IC2. The most common ST in IC2 was ST195 with 30 isolates, but 32 different STs made up IC2, making it a very diverse group. A proportion of CRAb isolates found in the Netherlands formed genetic clusters with international isolates, had known links to foreign countries, and were related to ICs or genogroups (GGs), indicating import in the Netherlands and further global dissemination of CRAb lineages. The presence of *bla*_NDM_ slightly increased between pilots 1 and 2 and should be closely monitored. Notably, the genomic epidemiology of CRAb in the Netherlands changed with the influx of male Ukrainian patients carrying CRAb since 2022. CRAb isolates from Ukrainian patients were genetically distinct from CRAb isolates from patients in the Netherlands, were mostly from IC2, showed distinct resistance gene profiles (*bla*_OXA-23_-like or *bla*_OXA-24_-like), and lacked *bla*_NDM_.

For both pilots, men represented 67% of the patients carrying or infected with CRAb. The characteristics of CRAb derived from Ukrainian patients were distinct from CRAb isolates found in patients from the Netherlands. The majority (96%) of the Ukrainian patients were male, and the median age of 36 is within the Ukrainian age of military service (25–55 years as of writing). The predominance of male patients with *A. baumannii* infections has been found in other studies as well (13, 18). The percentage of Dutch patients known to have been treated in a foreign hospital less than 2 months before culture sampling increased from 27% to 39% between pilot 1 and 2, and they had mostly been hospitalized in Morocco, Greece, and Türkiye. Other countries of note were Thailand, Indonesia, and China. This demonstrates that foreign hospitalization in countries where CRAb is prevalent remains an important risk factor for colonization or infection with CRAb in the Netherlands. It should be noted that due to the setup of the questionnaire, there were 126 unknown answers regarding foreign hospitalization. This question was optional during data collection and was not always answered. The percentage of patients known to have been treated in a foreign hospital is therefore the minimum known amount. All other questions were mandatory and gave an accurate representation of the epidemiology. There was also a larger proportion of CRAb isolates from Ukrainian patients obtained from wounds (29% Ukrainian vs 16% Dutch patients), possibly because this group is largely composed of war casualties. Notably, we found no clusters containing isolates from both Ukrainian and Dutch patients, suggesting that the Dutch preventive screening and isolation policy in hospitals may successfully prevent further transmission of CRAb.

We found 26 genetic clusters among the 204 isolates of this study. Out of these 26 clusters, 4 clusters contained only patients who had been hospitalized in Ukraine, and 15 clusters contained patients who had been hospitalized before in another foreign country. There were no data concerning hospitalization in a foreign country for the remaining seven clusters. Most isolates and clusters were found in IC2, which represents a global disseminated lineage and was made up of 32 different STs (2, 13–15, 19). In addition, we found a large genetic cluster containing isolates originating from 13 countries, including three isolates from patients from the Netherlands with no known foreign hospitalization. These three CRAb isolates from Dutch patients did not form a genetic cluster among themselves and differed by >28 AD. This suggests that either other related CRAb isolates were not detected by the surveillance, did not spread in the Netherlands, or represented cross-border clonal spread. We found three clusters with a link to Morocco in IC9 (STs 1,089, 1,841, and 2,782), and IC9 is mostly linked with Southern Spain, Saudi Arabia, and Tunisia (20–22). While IC9 isolates are less prevalent than IC2 in this study (23× IC9 vs 126× IC2), 74% of IC9 isolates carried a *bla*_NDM-1_ gene, compared to 10% of isolates in IC2 (10%). Isolates of IC9, and especially ST1089 and ST2782, therefore seem more likely to contribute to the spread of *bla*_NDM-1_.

We analyzed CRAb isolates assigned to a certain IC and, using wgMLST, observed that isolates belonging to the same IC group together with a distance of less than 1,000 AD and a distance of >1,000 AD between the ICs. Most strains within an IC did not form a genetic cluster, so these isolates were not clonally related and have divergent genetic backbones. Therefore, it might be more correct to define isolates with an AD of less than 1,000 as a GG instead of using the IC nomenclature for the international analyses.

CRAb analyzed in this study showed genetic markers associated with beta-lactam for all isolates and aminoglycoside resistance in >95% of isolates. For all other antibiotic resistance classes, CRAb isolates from Ukrainian patients were different. Genes associated with resistance to amphenicol, folate pathway antagonists, and rifamycin were more often present in CRAb isolates from Ukrainian patients compared to the isolates from Dutch patients. Conversely, macrolide resistance and especially tetracycline resistance genes were less present in isolates from Ukrainian patients.

With the exception of aminoglycoside and beta-lactam resistance genes, the majority of resistance genes in this study were localized on the chromosome. The proportion of aminoglycoside and beta-lactam resistance genes found on at least one plasmid has increased between pilots 1 and 2. This suggests that while many of the CRAb isolates also carry aminoglycoside and beta-lactam resistance genes on the chromosome, the presence of a plasmid carrying these resistance genes may drive the spread of antibiotic resistance. *bla*_NDM-1_ was identified on a plasmid only once, whereas it was localized on a chromosome 18 times. It is possible that these findings indicate that there is limited spread of *bla*_NDM-1_ carrying plasmids among CRAb in the Netherlands, but it could also suggest that *bla*_NDM-1_ carrying plasmids are rapidly incorporated into the chromosome. Mobile genetic elements and recombination of genes are known to play an important role in *A. baumannii* (23). Other antimicrobial resistance mechanisms do exist, such as the loss of efflux pumps and porins, or mutation of antibiotic target sites, but these were not analyzed in this study.

Taken together, these findings highlight the importance of genomic surveillance of CRAb, as this provides insight into the potential routes through which different CRAb lineages and carbapenemase genes spread in the Netherlands. Continued screening for CRAb in patients with known foreign hospitalizations is important, and healthcare professionals should be aware of the increasing occurrence of NDM-type carbapenemases in CRAb.

## MATERIALS AND METHODS

### Selection of CRAb isolates in 2015–2017 and 2022–2024 and phenotypical tests

A retrospective genomic surveillance study was performed using data of the Dutch national CPAb pilot in the period January 2015 until December 2017 (termed pilot 1) and the CRAb pilot surveillance in the period August 2022 until July 2024 (pilot 2). While pilot 1 was a CPAb pilot surveillance, only two isolates did not meet the CRAb criteria (EUCAST v15.0, MIC meropenem >8 mg/L). Since pilot 2 was a CRAb pilot surveillance, the criteria of pilot 2 were chosen for CRAb isolate inclusion in this study. CRAb isolates (MIC meropenem >8 mg/L) were requested from Dutch medical microbiology laboratories (MMLs) using the national Type-Ned surveillance system. Received *A. baumannii* isolates were identified using matrix-assisted laser desorption/ionization time-of-flight (MALDI-TOF), and carbapenemase production of all isolates was tested with the CIM in the RIVM (24). The MIC for meropenem was determined with E-test (BioMerieux Inc., Marcy L’Etoile, France). Another selection criterion was the presence of a 128-character encrypted personal identification number to deduplicate isolates from one person per pilot group. We also included CRAb isolates collected from patients who originated from Ukraine and were hospitalized and sampled in the Netherlands during pilot 2, without the encrypted personal identification number, as it was previously noted that hospitalization in Ukraine formed an important risk factor (25). Therefore, pilot 2 was divided into two groups, CRAb isolates originating from patients from the Netherlands (defined by having a unique encrypted personal identification number) and isolates originating from patients from Ukraine.

### Meta data and isolate collection

Isolates were collected from all MMLs through the national Type-Ned surveillance system for carbapenemase-producing microorganisms, which covers the majority of the healthcare institutions of the Netherlands. Here, participating MMLs can submit carbapenemase-producing microorganisms of interest (such as CRAb) along with epidemiological data to be characterized and sequenced pseudo-anonymously by the RIVM (17, 26). Submitted CRAb isolates were detected either during the identification of infections or during screening. In the Netherlands, hospital guidelines recommend screening for carbapenemase-producing microorganisms if a patient was transferred from a foreign hospital, hospitalized in a foreign hospital less than 2 months ago for more than 24 h, underwent an invasive procedure in a foreign hospital less than a year ago, or lived in an asylum center less than 2 months ago.

Clinical and epidemiological data were extracted from the Type-Ned surveillance database (27). These included questions on the reason for culturing, the type of healthcare setting for sampling, colonization or infection, risk factors such as underlying disease, hospitalization abroad, and contact with animals, and geographic data. Participating MMLs filled out these data based on available information in the local laboratory information system and electronic patient medical records. In cases where optional data were missing, such as for foreign hospitalization, the data were interpreted as not available, and no assumptions were made about hospitalization.

### Whole-genome sequencing of CRAb

All isolates were whole-genome sequenced with both Illumina next-generation sequencing and Oxford Nanopore Technologies long-read third-generation sequencing. In brief, for pilot 1, CRAb isolates were subjected to WGS using the Illumina HiSeq 2500 (BaseClear Leiden, the Netherlands) (26), and for pilot 2, CRAb were sequenced using the Illumina NextSeq550 platform (Illumina, USA), according to the manufacturer’s instructions as described previously (17). Long-read sequencing was performed for pilot 1 as described in reference 26 and for pilot 2 as described in reference 17. For WGS data from both pilots, read quality analysis and *de novo* assembly were performed using the Juno-assembly v2.0.2 pipeline from the RIVM (https://github.com/RIVM-bioinformatics/juno-assembly). Both Illumina and Nanopore data were used in a hybrid assembly performed by Unicycler v0.5.0. Illumina data were not trimmed before running Unicycler, which was operated using default settings and verbosity 2. The resulting contig files were annotated using Bakta v1.9.2 and were subsequently loaded into BioNumerics v8.1.1 (BioMérieux) for further analysis (28). MALDI-TOF cannot distinguish the different *Acinetobacter* species within the ABC-complex properly. Therefore, *Acinetobacter* species was also determined based on WGS data using KRAKEN2 v2.1.3 (29). Additionally, we downloaded WGS data from 577 isolates from three different studies from NCBI, which were selected because of their high genetic and geographic diversity and high quality. The NCBI data were processed the same way as the WGS data from this study. The WGS data set (*n* = 577 total) included a large study from the United States (*n* = 137) (30), a study from Denmark (*n* = 133) (13), and a worldwide CRAb data collection study (*n* = 307) (14).

### Molecular typing and identification of antibiotic resistance genes

All isolates were typed with MLST (Oxford and Pasteur schemes) (31, 32) and wgMLST with specific schemes for *A. baumannii* (33), using SeqSphere+ version 9.0.8. Unidentified STs were uploaded to the PubMLST website (http://pubmlst.org/abaumannii/) for the assignment of new STs. ICs were assigned using a cutoff of <1,000 wgMLST AD. To identify antibiotic resistance genes in the assembled contigs, a conda database (https://genepi.food.dtu.dk/resfinder/) with ResFinder (v4.1.11) and PlasmidFinder (V2.1.6) from the Center for Genomic Epidemiology was used. A threshold of 95% was used for identity and 60% for length. For the detection of the correct variant for *bla*_OXA_ genes, AMRFinder (V3.11.11) was used with the same thresholds in case of no 100% identity with a reference from the ResFinder database. For the identification of chromosomes or plasmids, only circular assemblies were used. Circular assemblies larger than 1 MB were assigned a chromosome, and circular assemblies between 2 kB and 500 kB were assigned as a plasmid.

## Data Availability

The sequence data generated and analyzed in this study are available in the Sequence Read Archive (SRA) under the following project: PRJNA1278965. Isolates published in previous works can be found under PRJNA903550 and PRJNA1076808. Sequence data used for international comparison were published previously under BioProjects PRJEB27899, PRJEB60981, and PRJNA667024. File S1 contains the NCBI accession and BioProject numbers of the Dutch CRAb isolates. The authors confirm that all supporting data, protocols, and accession numbers have been provided within the article and through supplemental files.

## SUPPLEMENTAL MATERIAL

Fig. S1 spectrum.02602-25-s0001.pdf Boxplot of the sum of antibiotic resistance classes present in CRAb from the Netherlands.

File S1 spectrum.02602-25-s0002.xlsx Bacterial isolates used in this study with metadata.

Supplemental figure legend spectrum.02602-25-s0003.docx Legend for Fig. S1.

ASM does not own the copyrights to Supplemental Material that may be linked to, or accessed through, an article. The authors have granted ASM a non-exclusive, world-wide license to publish the Supplemental Material files. Please contact the corresponding author directly for reuse.
