# Supplementary figures and images for: Comparative genomic surveillance of carbapenem-resistant Acinetobacter baumannii in the Netherlands in 2015–2017 and 2022–2024
Source: Microbiol Spectr. 2026 Feb 26;14(4):e02602-25. doi: 10.1128/spectrum.02602-25 (PMC13055278; doi:10.1128/spectrum.02602-25)

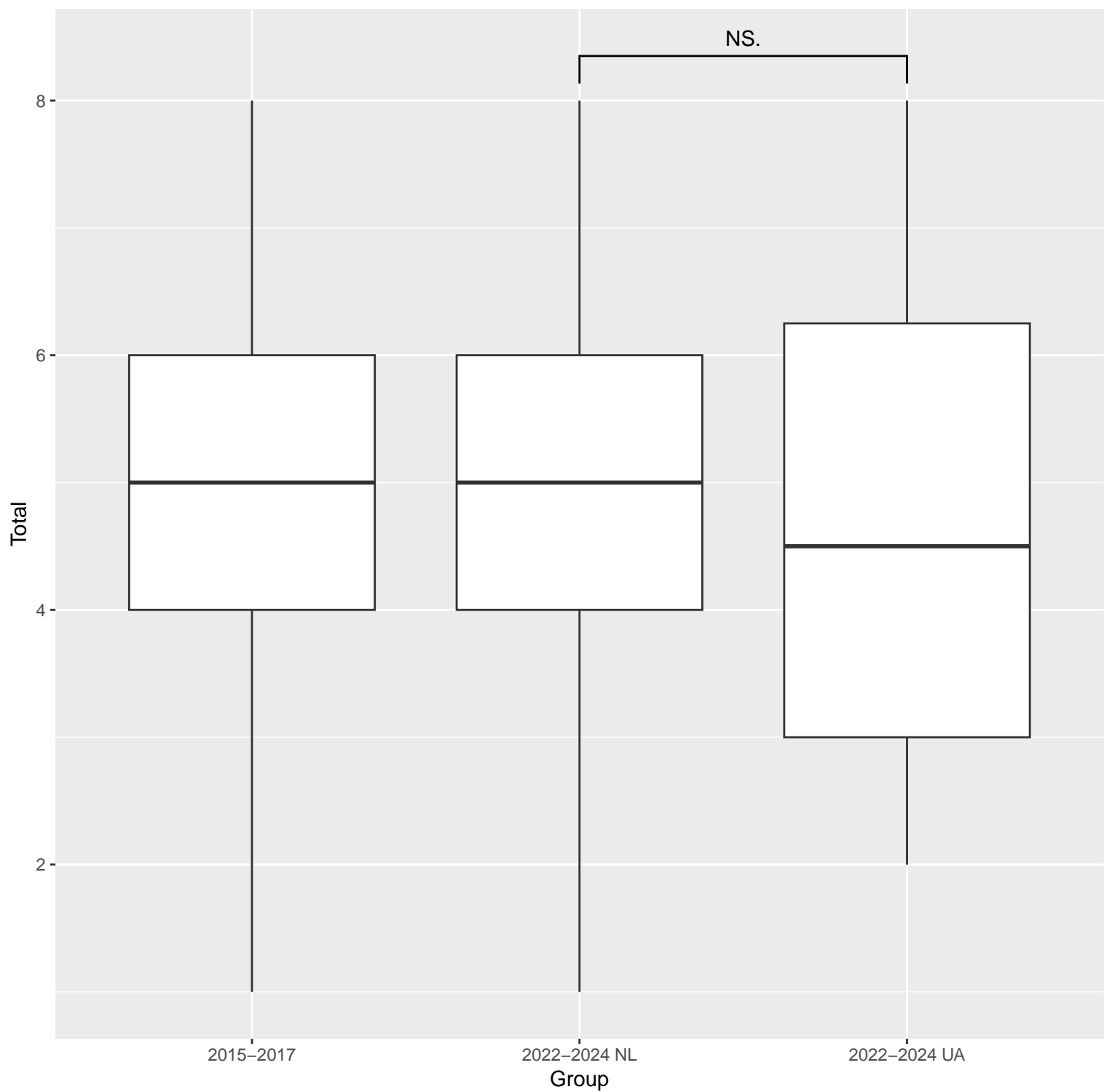

Supplement: Fig. S1 — Boxplot of the sum of antibiotic resistance classes present in CRAb from the Netherlands. [file spectrum.02602-25-s0001.pdf]
