# Supplementary material for: Comparative genomic surveillance of carbapenem-resistant Acinetobacter baumannii in the Netherlands in 2015–2017 and 2022–2024
Source: Microbiol Spectr. 2026 Feb 26;14(4):e02602-25. doi: 10.1128/spectrum.02602-25 (PMC13055278; doi:10.1128/spectrum.02602-25)
Supplement: Supplemental figure legend — Legend for Fig. S1. [file spectrum.02602-25-s0003.docx]

Fig. S1 Resistances towards antibiotic classes
